# Risk factors for limited improvement after total trapeziometacarpal joint arthroplasty

**DOI:** 10.1186/s12955-020-01333-z

**Published:** 2020-03-30

**Authors:** Sebastian Breddam Mosegaard, Maiken Stilling, Torben Bæk Hansen

**Affiliations:** 1 University Clinic for Hand, Hip and Knee Surgery, Department of Orthopaedics, Lægårdvej 12, 7500 Holstebro, Denmark; 2 Department of Clinical Medicine, Aarhus University, Palle Juul-Jensens Boulevard 99, Aarhus N, 8200 Denmark

**Keywords:** Osteoarthritis, Trapeziometacarpal joint, Total joint replacement, Risk factors, Functionality, Postoperative improvement

## Abstract

**Background:**

Trapeziometacarpal (TMC) osteoarthritis can be painful and cause disability for patients. Total joint replacement of the TMC joint provides a pseudo arthrosis with good restoration of the thumb motion and pain relief in most patients. But there is also a risk of no improvement following the operation. The purpose of this study was to identify patients at risk of no clinically important improvement following operative treatment of osteoarthritis of the TMC joint.

**Methods:**

We included 287 consecutive patients (225 women, 62 men) treated with total joint replacement of the TMC joint due to osteoarthritis with a mean age of 58.9 years (range 41–80) in a prospective cohort study. We collected information preoperatively and 12 months postoperatively on disabilities of the arm, shoulder and hand score (DASH), grip strength and pain at rest and activity on a visual analogue scale (VAS).Results: We found a statistically significant improvement in DASH from 42.0 to 15.9 (*p* < 0.001), VAS at rest from 3.5 to 0.6 (*p* < 0.001), VAS at activity from 7.9 to 2.5 (*p* < 0.001) and grip strength from 21.6 kg to 27.6 kg (*p* < 0.001) 12 months after the operation, when analysed as a group. There was an increased risk of no clinically important improvement in hand function for patients with preoperative high preoperative grip strength. Also, we found an increased risk of no clinically important improvement in female patients when using VAS as outcome.

**Conclusion:**

However, we were unable to detect one isolated preoperative predictor as indicator of successful result after operative treatment of TMC osteoarthritis, and as so it was not possible to establish a clinical valid tool for patient selection before surgery.

Informed consent was obtained from all patients for being included in the study. The study needed no approval from The Regional Committee of Biomedical Research Ethics as the data was collected, as part of our normal pre- and postoperative clinical pathway, but the study is part of an outcome study of the results after total joint arthroplasty (TJA) of the TMC joint registered in Clinicaltrials.gov (NCT01554748).

**Trial registration:**

Clinicaltrials.gov (NCT01554748). Registered 15 March 2012.

## Introduction

Osteoarthritis of the trapeziometacarpal (TMC) joint is a very common condition with a prevalence of more than 40% in men and women older than 50 years [1] leading to impaired hand function with pain and reduced grip and pinch strength. The standard operative treatment after failed conservative treatment is trapeziectomy with or without interposition arthroplasty [2]. Trapeziectomy provides a pseudoarthrosis with good restoration of thumb motion and pain relief in most patients where up to 86% would undergo the same surgery again [3].

Total joint replacement of the TMC joint has also been used for years as treatment of TMC joint osteoarthritis. The implant design is a ball and socket articulation resembling a total hip arthroplasty, with a metacarpal stem and modular neck-head segment which articulates with a trapezium cup. The first TMC implants were cemented [4], but during the last 10–15 years cementless TMC implants have been widely introduced, and improvements in cup and stem designs have increased implant survival [5–7].

Total joint replacement of the TMC joint may give a more rapid rehabilitation and better restoration of grip and pinch strength compared to Trapeziectomy [8, 9]. However, careful patient selection and information is important due to a relatively high risk of complications leading to the need for revision surgery with a possible salvage procedure and removal of the implants [10].

In recent years, a general treatment effect-measure of surgical hand intervention, which include the value for the patient, has been debated [11], but not yet defined [12, 13].

The purpose of this study was to see if it is possible preoperatively to identify patients at risk of no clinically important improvement in hand function or symptoms after operative treatment of osteoarthritis with total joint replacement of the TMC joint based on a statistical prediction model using preoperative assessments, and to establish a combination of patient reported outcome measures to be used in evaluation of the result after operative treatment of osteoarthritis of the TMC joint.

## Methods

The study is based on a consecutive cohort of 375 hands in 287 patients (79% female hands, *n* = 298) with a mean age of 58.7 years (range 41–80) treated for osteoarthritis in the TMC joint using TMC TJA in the period 2008–2015 at the Department of Orthopaedics at Holstebro Regional Hospital. Patients were treated with six different prosthesis models (Table 1). The treatment was carried out by a small team of 4 surgeons using the same indications and treatment protocol throughout the study period.

**Table 1:** Showing the number and percentage of patients treated with different prosthesis in this study. Furthermore, the table shows the different baseline characteristics within each prosthesis group with mean and 95% confidence intervals

Prosthesis	1, *N* = 62	2, *N* = 142	3, *N* = 10	4, *N* = 41	5, *N* = 20	6, *N* = 12
	Mean (95% CI)	Mean (95% CI)	Mean (95% CI)	Mean (95% CI)	Mean (95% CI)	Mean (95% CI)
DASH	37.5 (32.9–42.1)	47.8 (44.2–51.3)	36.7 (29.7–43.7)	36.6 (30.4–42.8)	34.3 (24.4–44.1)	45.7 (29.6–62.8)
VAS activity	7.7 (7.2–8.2)	8.2 (7.9–8.5)	7.1 (5.2–9.0)	7.9 (7.3–8.5)	7.5 (6.5–8.6)	8.6 (7.2–9.8)
VAS rest	3.5 (2.9–4.1)	3.8 (3.4–4.3)	3.0 (1.4–4.6)	3.1 (2.3–3.9)	2.9 (1.7–4.1)	4.5 (3.5–5.5)
Grip strength	24.1 (20.5–27.7)	20.8 (18.8–22.7)	25.9 (15.6.- 36.1)	22.8 (17.6–28.0)	22.7 (15.2–30.2)	16.3 (5.9–26.8)
Age	58.8 (56.3–59.8)	58.5 (57.2–59.8)	57.9 (51.2–64.6)	60.5 (58.6–62.5)	60.0 (56.7–63.3)	60.4 (54.6–66.2)

Prosthesis 1 = Elektra Bimetal cementless cup, prosthesis 2 = Moovis press-fit dual-mobility cementless cup, prosthesis 3 = Elektra cemented polyethylene cup, prosthesis 4 = Motec cemented polyethylene cup, prosthesis 5 = Motec cementless titanium cup, prosthesis 6 = Elektra cementless cup. All patients were treated with ball and socket design prosthesis with different cup designs combined with cementless titanium metacarpal stems. DASH = The disabilities of the arm, shoulder and hand. Grip strength is measured in kg

Nine patients were excluded due to missing 12 months follow-up. All the nine patients (2.5%) had a reoperation with trapeziectomy during the first 12 months postoperative. In two patients the reason was an undiscovered intra-operative trapezium fracture, in 4 patients the reason was postoperative trapezium fracture after thumb trauma, in 1 patient multiple joint dislocations, and in 2 patients the reason was a cementing failure leading to lack of cup fixation. In the study period we used TJA as standard treatment in patients with symptomatic Eaton grade 2–3 osteoarthritis of the TMC joint. Trapeziectomy was only used in patients with Eaton grade 4, patients with severe comorbidity and patients not willing to have the risk of TJA implant failure.

We collected data on disabilities of the arm, shoulder and hand (DASH), pain at rest (VAS at rest), pain at activity (VAS at activity) and grip strength prospectively. DASH and VAS was collected using a self-reported questionnaire. The DASH questionnaire is a 30-item questionnaire used to measure patient reported disability through 30 statements on a 5-point Likert scale, where a higher score reflects more disability. The total score was then transformed to a score out of 100 by subtracting one and multiplying by 25. Grip strength was measured by an independent observer (outpatient clinic nurse) using a dynamometer (Jamar hand dynamometer, North Coast Medical, Morgan Hill, CA).

We did not have any specific inclusion criteria and included all patients having a total joint replacement of the TMC joint due to osteoarthritis.

To avoid statistical dependence only the first operated hand was included in bilateral operated patients, leaving 287 hands/patients with a mean age of 58.9 years (range 41–78) and consisted of 78% females (*n* = 225). The patients were followed prospectively with self-reported pain score at rest and activity (VAS from 0 to 10) with a higher score indicating higher pain, grip strength (kg) and DASH with a higher score indicating higher disability [14] preoperatively and after 12 months. We used a Danish translated and validated version of the DASH questionnaire [15, 16].

The procedures followed in this study were in accordance with the Helsinki Declaration of 1975, as revised in 2000. The study was generally approved by the local research ethics committee, and no further specific approval was demanded because the study is an outcome study, which according to the Danish law “Act on a Biomedical Research Ethics Committee System and the Processing of Biomedical Research Projects”, Part 3 “Notification and authorization”: Questionnaire-based projects and register research projects shall only be notified to a regional committee if the project also involves human biological material. The study was registered in The Danish data Protection Agency and ClinicalTrials.gov Identifier: (NCT01554748).

## Statistical analysis

Logistic regression and linear regression models were used to test predictors of patient reported outcome in VAS, DASH and grip strength. Dichotomous dependent variables were required for logistic regression, and these were defined by the change in VAS, DASH and grip strength from preoperative measurements to measurements made 12 months postoperatively. A previous study found the minimal clinical important difference (MCID) for DASH to be ten points (range 5–15) [17]. The MCID for the Danish version of DASH has been found to be 12 points [18]. We defined a positive change in DASH to be a postoperative DASH reduction > 15 points lower than the preoperative, which should secure that a positive outcome is really clinically important. Also, a change in DASH of 15 points is recommended by the DASH organization on their website as limit for registration of changes. For a positive change in pain at activity and rest, the postoperative measurement was set to be > 2 VAS points lower than preoperative as the MCID [19]. Based on a previous study that found the clinically important difference in grip strength to be 19%, we defined a positive change to be a postoperative measurement > 19% higher than the preoperative [20]. Additionally, two new combined variables VAS rest + DASH and VAS activity + DASH were defined. A positive outcome was defined by a positive outcome in both VAS at rest and DASH or VAS at activity and DASH respectively. According to Peduzzi et al., [21] the sample size using a multiple logistic regression model can be estimated using the formula “N = (10 * covariates)/ smallest proportion of success failure”. This estimates a sample size of 250 for our most demanding regression model. Using GPower software we conducted a sensitivity analysis for the required effect size with a = 0.05, power = 0.8 and sample size = 287 showing a required effect size of odds ratio = 1.52.

Collinearity in the regression model was inspected using variance inflation factor (VIF) showing VIFs ranging from 1.06 to 2.17, revealing no critical collinearity problems. In both Tables 2 and 3 the same potential predictive covariates were used, including: Preoperative VAS at rest and activity, preoperative DASH score, preoperative grip strength, prosthesis type, age and gender. Since we were unable to identify prediction studies on total joint replacement in the TMC joint, the decision on variables included was made from existing literature on other hand related prediction studies. The tested predictive variables with a *p* value > 0.09 are not presented in the tables as these are far from being statistically significant.

**Table 2:** Adjusted odds ratios for independent variables included in the multiple logistic regression model for prediction of positive outcome

Outcome	Adjusted Odds ratio	95% CI	*p* value
VAS at activity			
Preoperative DASH	0.96	0.94–0.99	0.002^*^
Preoperative Grip strength	0.96	0.91–1.00	0.048^*^
Male vs female ref	3.53	0.96–12.97	0.057
VAS at rest			
Preoperative grip strength	0.96	0.92–1.01	0.088
Male vs female ref	4.12	1.19–14.22	0.025^*^
DASH			
Preoperative grip strength	0.96	0.92–1.01	0.086
Grip strength			
Male vs female ref	0.50	0.23–1.09	0.081
DASH + VAS at rest			
Preoperative grip strength	0.95	0.91–0.99	0.044^*^
Male vs female ref	2.77	0.86–8.93	0.088
DASH + VAS at activity			
Preoperative grip strength	0.95	0.92–0.99	0.044^*^

The table is divided into the six different outcome measures: “VAS at activity”, “VAS at rest”, “DASH”, “Grip strength”, “DASH + VAS at rest” and “DASH + VAS at activity”. DASH = The disabilities of the arm, shoulder and hand. Grip strength is measured in kg. All models are adjusted for baseline measurements, prosthesis, age and gender. Predictors with a *p* value > 0.09 are not presented in the table

^*^Indicates a significant *p*-value below 0.05

**Table 3:** Coefficients for independent variables included in the multiple linear regression model for prediction of improvement in VAS at rest and activity, grip strength and DASH score

Outcome	Coefficient	S.E.	95% CI	*P* value
VAS at activity, *R*^*2*^ = 0.08				
Preoperative DASH score	−0.05	0.01	−0.08 - -0.02	0.001^*^
Male vs female ref	1.73	0.75	0.25–3.20	0.022^*^
VAS at rest, *R*^*2*^ = 0.13				
VAS at activity	0.28	0.10	0.09–0.47	0.004^*^
Preoperative DASH score	0.02	0.01	0.00–0.04	0.020^*^
Preoperative grip strength	−0.05	0.02	− 0.09 - -0.01	0.009^*^
Male vs female ref	1.14	0.56	0.05–2.24	0.043^*^
DASH score, *R*^*2*^ = 0.07				
Preoperative grip strength	−0.42	0.16	−0.73 - -0.11	0.009^*^
Grip strength, *R*^*2*^ = 0.01				
Preoperative VAS at rest	0.53	0.30	−0.08 – 1.10	0.088

The table is divided into four different outcome measures: “VAS at activity”, “VAS at rest”, “DASH” and “Grip strength”. DASH = The disabilities of the arm, shoulder and hand. Grip strength is measured in kg All models are adjusted for baseline measurements, prosthesis, age and gender. Predictors with a *p* value > 0.09 are not presented in the table

^*^Indicates a significant *p*-value below 0.05

Patients with missing data on all variables were excluded as it was not possible to calculate a difference/improvement score. Patients with partially missing data were only used to calculate overall mean postoperative improvement.

Further, to avoid ceiling effect patients with preoperative DASH< 15 and VAS < 3 were not used in the logistic regression analysis of preoperative predictors of outcome. With regards to the external validity of the results from the logistic regression models these should only be related to patients with preoperative scores above or equal to the MCIDs for DASH (15) and VAS (3). When using DASH as outcome 13 patients were excluded due to too low preoperative DASH. When using VAS at rest and VAS at activity as outcome 82 and 2 patients respectively were excluded due to too low preoperative VAS. No patients had preoperative DASH< 15 and VAS at activity< 3. These excluded patients were only used when calculating pre- and postoperative mean scores. The tests of differences between pre- and postoperative mean scores was made using Wilcoxon signed rank test. A significance level of 0.05 was used in all models. All statistical analyses were made using STATA, version 15 IC (Stata Corp, College Station, TX, USA).

## Results

**Fig. 1:**
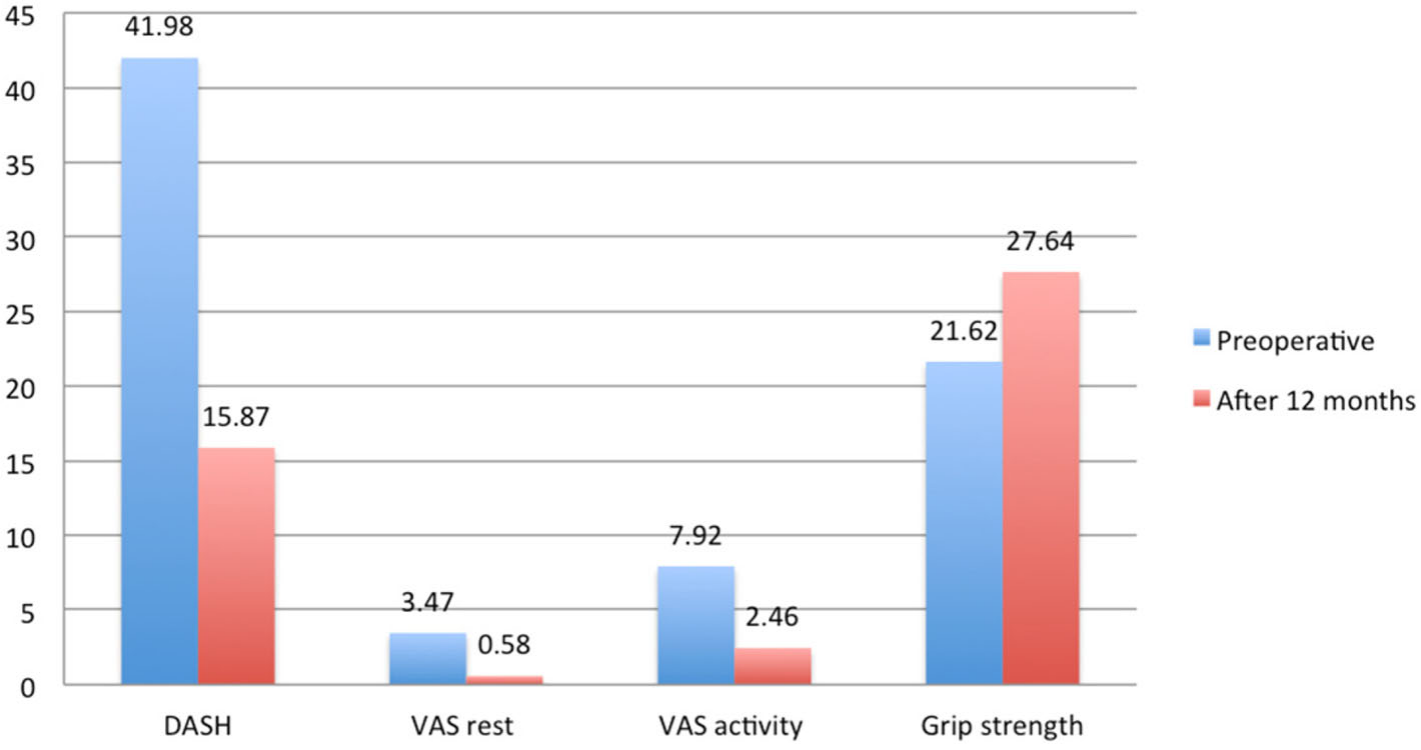
Preoperative and 12 months postoperative measurements. Legend: Showing mean scores for DASH, VAS at rest, VAS at activity and grip strength before - and 12 months after total joint replacement of the trapeziometacarpal joint. There were significant improvements in all four measures (*p* < 0.001)

Overall, we found a statistically significant improvement in DASH, VAS and grip strength 12 months after the operation, when the patients were analysed as a group. The mean grip strength was 21.6 kg (SD 12.2) preoperatively and 27.6 kg (SD 12.0) postoperatively, with a mean improvement in grip strength of 6.0 kg (SD 9.0) (*p* < 0.001). The mean DASH score was 42.0 (SD 18.6) preoperatively and 15.9 (SD 17.5) postoperatively, with a mean improvement in DASH score of 26.1 (SD 18.50) (*p* < 0.001). The mean VAS at rest was 3.5 (SD 2.4) preoperatively and 0.6 (SD 1.4) postoperatively, with a mean improvement in VAS at rest of 2.9 (SD 2.5) (*p* < 0.001). The mean VAS at activity was 7.9 (SD 1.8) preoperatively and 2.5 (SD 2.8) postoperatively, with a mean improvement in VAS at activity of 5.4 (SD 3.1) (*p* < 0.001), (Fig. 1).

**Fig. 2:**
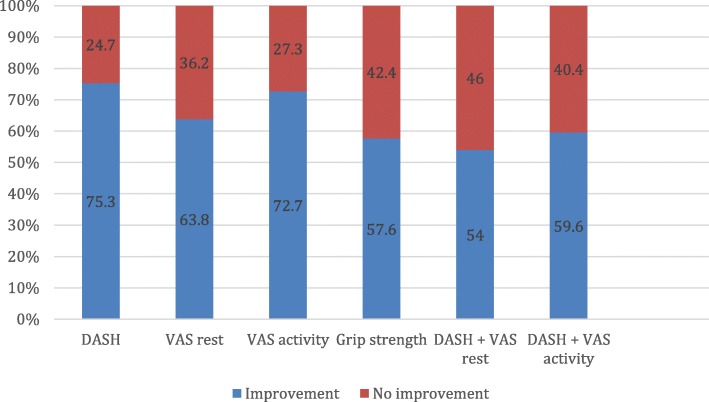
Percentage of patients improving 12 months postoperatively. Legend: Percentage of improvement and no improvement defined by different outcome variables (DASH, VAS at rest, VAS at activity, grip strength, DASH + VAS at activity and DASH + VAS at rest)

The percentage of successful joint arthroplasties based on the MCIDs are shown in (Fig. 2).

The predictive variables were not the same among the different outcome variables.

### VAS as outcome variable

Using VAS at activity as outcome, higher preoperative DASH (*p* = 0.001) and higher preoperative grip strength (*p* = 0.048) decreased the probability of a clinically important improvement, (Table 2). McFadden’s pseudo *R*^*2*^ for this model was 0.10. We found that approximately 50% of patients with preoperative VAS at activity from 3 to 6 (*n* = 41) did not reach a clinically important improvement using VAS at activity as outcome measure.

Using VAS at rest as outcome, male gender (*p* = 0.025) increased the probability of a clinically important improvement, (Table 2). McFadden’s pseudo *R*^*2*^ for this model was 0.06.

### Grip strength as outcome variable

Using grip strength as outcome, none of the explanatory variables had a significant effect, (Table 2). McFadden’s pseudo *R*^*2*^ for this model was 0.04.

### DASH as outcome variable

Using DASH as outcome, none of the explanatory variables had a significant effect, (Table 2). We found that approximately 50% of patients with a preoperative DASH score between 15 and 24 did not reach a clinically important improvement. McFadden’s pseudo *R*^*2*^ for this model was 0.05 indicating that the model explains little of the variation in outcome. We carried out this analysis with an improvement in DASH score > 11 defining positive outcome as found to be the Danish validated MCID [18] and found no difference.

### VAS and DASH as combined outcome variables

Using the VAS rest + DASH variable as outcome, higher preoperative grip strength (*p* = 0.022) decreased the probability of a clinically important improvement, (Table 2). McFadden’s pseudo *R*^*2*^ for this model was 0.03.

Using the VAS activity + DASH variable as outcome, higher preoperative grip strength (*p* = 004) decreased the probability of a clinically important improvement, (Table 2). McFadden’s pseudo *R*^*2*^ for this model was 0.06 indicating that the model explains little of the variation in outcome. Furthermore, we found a correlation of (r = 0.3365) between preoperative measures of pain at activity and rest indicating that the patients did not interpret both questions alike.

Using multiple linear regression models, we examined the same covariates using absolute change values as dependent variables (Table 3). With these models we were still unable to identify find predictors for improvement in grip strength. Using VAS at activity DASH remained a predictor, whereas grip strength became insignificant and male gender were related to higher improvement (*p* = 0.022). Using VAS at rest men remained likely to improve more than women. Furthermore, both patients with higher preoperative DASH (*p* = 0.020) and higher VAS at activity (*p* = 0.004) were related to higher postoperative improvement. A higher preoperative grip strength was related to less postoperative improvement (*p* = 0.009). Higher preoperative grip strength was also related to less postoperative improvement using DASH score as outcome (*p* = 0.009).

## Discussion

We found a general improvement in both VAS at rest, VAS at activity, DASH and grip strength after operation for osteoarthritis in the TMC joint with a total joint replacement. When using the defined MCIDs in improvement as outcome we found that 25–46% of patients did not improve (Fig. 2) and that the predictive effect of baseline measurements varied. It was not possible to identify one specific preoperative measure that had a significant effect on all outcome measures.

In this study, we only used patients treated with TJA, because this type of treatment is the standard in our clinic in this type of patients. This choice of treatment is controversial due to high failure rates, but the failure rate during the first 12 months is very low (2.5%) and may not have biased the outcome evaluation. Furthermore, the rapid restoration of movement and grip strength after total joint TJA leads to overall improvements that make a good base for analysis in this outcome study. We did not test for difference in outcome between the different prosthesis since we believe that the short-term effect within 12 months does not vary between different implants but first occurs later due to different designs of the implants resulting in different failure rates over time. We did however adjust for prosthesis type in the logistic- and linear regression models to be sure that prosthesis type did not introduce bias.

Unfortunately, similar studies of the effect of operation due to carpometacarpal osteoarthritis have to our knowledge not been made. However, the effect of surgery on other hand conditions have been studied, especially the effect of Carpal Tunnel Release (CTR) and surgical treatment of Distal Radius fractures (DRF). Female gender has a tendency to increase the risk of no clinically important improvement in CTR [22] and surgical treatment of Distal Radius fractures [23]. Also, females are more likely to develop Chronic Pain Syndrome (CRPS) following surgical treatment of DRF [24, 25], with an estimated odds ratio of 3 to 4 [26]. However, other studies did not find predictive effect of gender after CTR [27] or on recurrence after Open Ganglion Excision [28]. Slutsky et al. proposed that these differences in the effect of gender on outcome might be due to differences in expectations, functional demands and pain tolerance between genders [29]. In our study we found an increased risk of nonclinical important improvement in pain at rest measured by VAS (Table 2). As our gender ratio is close to 1:4 this might affect the findings from this analysis.

We also found that older age at the time of operation negatively influenced postoperative VAS at rest and grip strength using multiple logistic regressions. In CTR the effect of age seems unclear as the results differ in different studies [22, 25, 27, 30, 31]. No effect of age has been found in studies on surgical treatment for DRF [23], surgical treatment for Dupuytren’s Contracture [32], and open dorsal wrist ganglion excision [28] which makes it hard to determine whether or not age at the time of operation has an effect on hand surgical outcome.

## Considerations and limitations

We used DASH, VAS at rest, VAS at activity and grip strength as outcome measures of successful TJA, but it may lead to some considerations and limitations.

### DASH score

The DASH outcome measure questionnaire includes questions about both arm, hand and shoulder disabilities. In this study, we examined the effect of TMC total joint replacement but other injuries and disabilities in the patient’s arm and shoulder can potentially influence the DASH improvement and lead to loss of validity. Additionally, some patients avoid answering certain personal questions from the DASH questionnaire, especially regarding sexual activities leading to missing responses with lack of basis for a total score and subsequently exclusion of 93 patients in the logistic regression analysis. We did not investigate the dominant hand involvements effect on the outcome.

Some questions evaluate tasks that are done with the dominant hand and not necessarily the injured hand making them difficult to answer and can potentially lead to bias. In our data 46% of patients had surgery on the left hand, which probably indicate that both dominant and non-dominant hands were treated. This might affect the validity of the DASH scores. However, the DASH questionnaire is not specifically targeting the operated hand, so the influence of hand domination may not be important.

Other measures of daily function might be more suitable than DASH. The Australian Canadian Osteoarthritis Hand Index (AUSCAN) is a hand specific osteoarthritis function score that do not relate to neither elbow nor shoulder [33] potentially eliminating bias due to comorbidities in elbow or shoulder. Additionally, the AUSCAN has a high reliability, is easily accessible and recommended for research use [33].

### VAS pain

To determine if patients improved in pain at rest and activity after the operation we used a VAS scale. When asking about pain at rest and activity we did not define a certain context. Thus, some patients might think of pain at rest as pain after finishing hand-demanding tasks while others might think of it as pain such as disturbing night sleep. The same potential problem of individual interpretation might affect pain at activity since the specific context is not explained. Due to the low correlation between pain at activity and pain at rest, we believe that patients were able to differentiate between pain at rest and pain at activity.

### Grip strength

There are several factors related to grip strength including both age and gender which we also found in the multiple logistic regression analysis. When considering age as predictor of outcome other factors than osteoarthritis in the TMC joint that can affect grip strength in older people. Patients could have other comorbidities we do not know about that could affect and minimize improvement in grip strength leading to lower validity. Furthermore, patients were measured using grip strength that examines the grip strength of the entire hand. As this study focuses on TMC arthritis pinch strength might have been more sensitive to changes in grip strength before- and after surgery.

### Combined DASH and VAS pain

We combined different outcome measures (DASH, VAS activity and VAS rest) to examine potential predictive preoperative factors in relation to treatment with TMC TJA but did not find a combination with higher predictive value than the single outcome models.

We found a high mean preoperative VAS at activity of 7.9 and a low mean preoperative VAS at rest of 3.5. Using a VAS MCID of 3 points we excluded multiple patients due to “too good” VAS at rest scores making it a difficult outcome measure. Due to the high pain score at activity we believe that VAS at activity should be used as outcome measure. Another important measure is hand function, which we measured using DASH. As previously described there are several limitations using DASH in relation to a hand specific surgery. It would probably have been better to use both pain at activity and a hand specific function score such as AUSCAN to evaluate the outcome following total joint replacement of the TMC joint.

Using our cut-off points for the combined DASH + VAS activity outcome we found that 40% did not reach a clinically important improvement. This could be explained by the surgery not being sufficiently effective, our cut-off points, or because some patients just had “too good scores” before surgery. Further, patients are often reporting either only high DASH score or high VAS score making improvement above the MCIDs for both DASH and VAS hard to reach.

In patients with a preoperative VAS at activity ranging from 3 to 6 (*n* = 41) we found that approximately 50% did not achieve a clinically important improvement using VAS at activity as outcome. Though only 41 patients had such low preoperative VAS at activity scores, it could indicate that some of the patients had “too good” VAS prior to surgery to achieve a clinically important improvement in VAS at activity.

Using different measures of outcome, we found low McFadden’s pseudo *R*^*2*^s indicating that other variables outside our models might increase the explanatory effect.

### Other potential predictors

Inclusion of other covariates as: work related factors, bone mineral quality, education and income would be of great interest. Other studies have found predictive effect of other preoperative measures such as education, income, smoking and alcohol use. In CTS patients low income [22], high alcohol consumption [31, 34] and smoking [34, 35] has been found to have a negative effect on surgical outcome. In DRF patients both low income [23, 24] and short education [24, 36] has been found to have a negative effect on surgical outcome. If we had asked about these prior to surgery we might have been able to explain more of the variability in outcome. Also, we did not ask about patient satisfaction, which would be an interesting outcome measure in order to examine the relationship between patient satisfactions, change in VAS, DASH and grip strength and preoperative measurements. We do not have data on patient’s analgesics use or patient expectation. It would be of great interest to include these as covariates in a future study.

The same study may have been performed in patients treated with trapeziectomy, but as this is not our preferred method, the number of trapeziectomies during the study period was very low, and the patients were not included in the study to avoid bias and confounding by indication.

## Conclusion

We were unable to detect one isolated preoperative predictor as indicator of successful result after operative treatment of TMC osteoarthritis, and as so it was not possible to establish a clinical valid tool for patient selection before surgery. Given that higher preoperative grip strength tends towards being a predictive factor in both the logistic- and linear regression models, patients with high preoperative grip strength might tend to improve less in both self-reported DASH and pain at rest and activity although not statistically significant in all models. When isolating a single outcome of interest this study shows that higher preoperative DASH and higher preoperative grip strength could be risk factors for nonclinical important improvement in pain at activity and combined DASH and pain respectively.

The surgeon should however be aware that patients with a preoperative high grip strength and females have an increased risk of having no clinical effect of the operation. Additional studies based on outcome of operative treatment of TMC joint osteoarthritis and patient satisfaction may provide greater explanatory power on potential preoperative predictors of outcome and help define a combined outcome of this surgical treatment.

## Data Availability

The datasets used and/or analysed during the current study are available from the corresponding author on reasonable request.

## Abbreviations

AUSCAN: The Australian Canadian Osteoarthritis Hand Index
CTR: Carpal tunnel release
CRPS: Chronic pain syndrome
DASH: Disabilities of arm, shoulder and hand
DRF: Distal radius fracture
MCID: Minimal clinical important difference
TJA: Total joint arthroplasty
TMC: Trapeziometacarpal
VAS: Visual analogue scale
